# Surviving at the highest and coldest: Nutritional and chemical components of fallback foods for Yunnan snub‐nosed monkeys

**DOI:** 10.1002/ece3.11219

**Published:** 2024-04-16

**Authors:** Hao Pan, Rong Hou, He Zhang, Yanpeng Li, Zhipang Huang, Liangwei Cui, Wen Xiao

**Affiliations:** ^1^ Institute of Eastern‐Himalaya Biodiversity Research Dali University Dali Yunnan China; ^2^ Shaanxi Key Laboratory for Animal Conservation, College of Life Sciences Northwest University Xi'an China; ^3^ International Centre of Biodiversity and Primates Conservation Dali University Dali Yunnan China; ^4^ Collaborative Innovation Center for Biodiversity and Conservation in the Three Parallel Rivers Region of China Dali Yunnan China; ^5^ Key Laboratory for Conserving Wildlife with Small Populations in Yunnan Southwest Forestry University Kunming China; ^6^ Yunling Black‐and‐White Snub‐Nosed Monkey Observation and Research Station of Yunnan Province Dali Yunnan China

**Keywords:** conservation, dietary selection, nutritional requirement, Yunnan snub‐nosed monkeys

## Abstract

Fallback foods (FBF), categorized into staple and filler types, are suboptimal food sources chosen by animals in response to a scarcity of preferred food items during specific periods. Using lichens as FBF by Yunnan snub‐nosed monkeys (*Rhinopithecus bieti*) represents a distinctive ecological adaptation and evolutionary development within nonhuman primates. This study delves into the annual dietary choices of the species to address issues, elucidate the nutritional value, and understand the ecological significance of lichens for this primate species, which resides at the highest altitudes and experiences the coldest weather among global primates. The findings reveal that the lichens consumed by the monkeys serve as the staple FBF, with *Bryoria* spp. and *Usnea longissima* being the primary dietary species. The former is the preferred choice, providing higher digestible fiber (neutral detergent fiber) levels but lower tannin, fat, ADF, and energy levels. During the dry season, lichens dominate as the monkeys' primary food and nutritional resources. In the wet season, they act as a fundamental food selection rather than an ideal dietary choice, substituting nutrients from fruits, seeds, and leaves. Compared to other Asian colobine counterparts, this species exhibits the highest lichen consumption but the lowest proportions of leaves, flowers, and seeds. This study provides valuable evidence and information for developing or amending conservation strategies and guidelines for the dietary management of captive breeding of monkeys, one of the world's critically endangered primate species.

## Graphical abstract

A family of Yunnan snub‐nose monkeys is enjoying their fallback food, lichen.

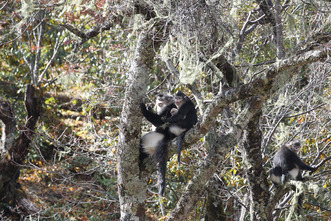

## 1 INTRODUCTION

Animals' dietary selection critically depends on external modifications in the environment, ecology, evolution, climate, habitat, air pollution, and internal population conspecific modifications in the dynamics of behaviors and interactions between populations or groups, which fluctuate periodically and seasonally. They, together, have affected animals' survivability and expect their development in the foreseeable future (Cao, 1989; Heiduck, 1997; Wang et al., 2020). Thus, understanding such external and internal changes is crucial for comprehending population ecology, evolutionary processes, and the overall functioning of ecosystems for a specific species. Thus, it is necessary to study alternative selection mechanisms, such as those revealing strategies to adopt different food resources and ecology (Litvaitis, 2000), and explore factors influencing such selection (Chapman, 1990). Such exertion can also help us identify the intensity of inter‐ and intra‐specific competition for food and analyze population division, fusion, and fluctuation affected by natural resources, environment, ecology, and habitats under the principles and regulations of natural selection and environment adaptation (Kirkpatrick, 1996; Van Schaik & Van Noordwijk, 1988). Such effort also enhances the understanding of other ecological aspects of animals for maintaining tangible conservation and management strategies (Liu et al., 2013).

Such exploration in nonhuman primates (primates thenceforth) has provided valuable information to understand their dietary preference, resource demand, and habitat‐carrying capacity (Grueter, Li, Ren, Wei, & van Schaik, 2009; Grueter, Li, Ren, Wei, Xiang, & van Schaik, 2009). It also offers guides to reconstruct and manage their habitat, improving their conservation status (Marshall et al., 2009).

Fallback food (FBF) is a low‐quality food resource chosen by animals due to the shortage of preferred food in a specific period (Marshall et al., 2009; Marshall & Wrangham, 2007). It has been considered to have two types: “staple” and “filler.” The former is consumed year around and seasonally can constitute up to 100% of the diet, and the latter never constitutes 100% (Marshall & Wrangham, 2007). Lichen consumed by *R. bieti* has been regarded as the FBF (Grueter, Li, Ren, Wei, Xiang, & van Schaik, 2009; Huang et al., 2017), a specific habitat‐dependent evolutionary selection.

Primates in temperate and cold regions face severe challenges because of low plant productivity in harsh habitats and chronic seasonal food shortages (Cramer et al., 1999; Grueter, Li, Ren, Wei, Xiang, & van Schaik, 2009; Latham & Ricklefs, 1993). They frequently adjust their feeding strategies to adapt to such challenges, including FBF selection (García‐Castillo & Defler, 2018; Grueter, Li, Ren, Wei, Xiang, & van Schaik, 2009; Harrison & Marshall, 2011). Current research on the FBF of primates focuses mainly on the definition, function, ecologic, and evolutionary adaptation of different species (Constantino & Wright, 2009; Grueter, Li, Ren, Wei, Xiang, & van Schaik, 2009; Laden & Wrangham, 2005; Lambert et al., 2004; Marshall et al., 2009; Marshall & Leighton, 2006; Marshall & Wrangham, 2007). Nevertheless, attention to nutritional and chemical components that differ among FBF species and present periodic variation seems to have not been seen. Their identification and amounts consumed from specific foods are gravely required to understand animals' specific demands to maintain a normal metabolism (Mattson Jr., 1980; Schoener, 1971), especially regarding protein, fat, fibers from which carbohydrate comes, and vitamins (Hale et al., 2018; Raubenheimer & Simpson, 2016). On the other hand, the exact demands for each item recorded from a given wild animal taxon provide essential guidelines for its captivity feeding (Hansell et al., 2020).

Among primates, colobines (Colobinae) food choices have been considered to primarily consist of leaves, fruits, flowers, and seeds (Huang et al., 2010; Kirkpatrick, 1996; Oates et al., 1980). The evolutionary development of Asian colobine ancestors from Africa to Asia during the Miocene and Plioce has dramatically shaped their morphology, ecology, and dietary selection (Zhang et al., 2022). Their phylogenetic development and distribution expansion in East and Southeast Asia have allowed them to adapt to alternative environments, ecology, and habitats and display a significant variation of altitudinal range from sea coastlines to more than 4000 m in Mt. Hengduan and the Qinghai‐Tibet Plateau, where *R. bieti* resides (Peng et al., 1993). Thus, this species is unique in colobines and the world's primates due to its specific adaptation to the harsh ecology surrounded by low temperature and hypoxia, besides scarce food resources (Grueter, Li, Ren, Wei, Xiang, & van Schaik, 2009). It is one of the critically endangered primate species on the Red List of IUCN (Long, Bleisch, & Richardson, 2020).

The frigid climate has made *R. bieti* require tremendous energy to maintain body temperature (Guo et al., 2018; Hou et al., 2020), especially during energy‐hungry periods in which food resources are scarce and variable due to seasonal climate changes, especially prolonged winter (Huang et al., 2012; Kirkpatrick, 1996; Van Schaik & Brockman, 2005). Thus, understanding how this primate species copes with such an environment and ecology through dietary selection and recording its nutritional and chemical components will provide valuable information and evidence for their conservation and captivity feeding before releasing them into the wild.

Therefore, the primary purposes of this study include: (1) elucidate what kind of FBF type the lichens consumed by *R. bieti* belong to; (2) understand how *R. bieti* is unique in evolutionary dietary selection compared with other Asian colobines; (3) understand the selective strategy of the lichen groups in terms of the nutritional and chemical components; and (4) provide scientific information in amending or making conservation strategies and guidelines for dietary management for the creatures in captivity feeding programs.

## 2 FIELDWORK AND MATERIAL

The study sites included five species groups in Mt. Lasha, from the south to the north of the species distribution, with an altitudinal variation between 2450 and 3600 m. The vegetation primarily consists of deciduous broad‐leaved forest, coniferous broad‐leaved mixed forest, and alpine dark coniferous forest from lower to higher altitudes. The distance between the groups is from 10 to 20 km. Each group's average home range, the daily activity range, is between 750 and 15,100 m. A typical group of the species consists of several one‐male and multi‐female breeding units (OMU) and one or two all‐male (bachelor) units (AMU).

Food types of the *R. bieti* include two lichen species (*Bryoria* spp. and *Usnea longissima*), three bamboo species (*Fargesia strigosa*, *Fargesia edulis*, and *Fargesia solida*), one species of mushroom (*Morchella esculenta*), and 29 other plant species (Appendix Table A1) (Huang et al., 2012). The average annual rainfall is 910 mm, 85% of which occurs from May to October, and the whole year can be divided into dry (November–April) and wet (May–October) seasons (Huang et al., 2012). The annual average temperature is 11.7°C, with the highest (17.4°C) in July and the lowest (−5.6°C) in February (Huang et al., 2012, 2017).

**FIGURE 1:**
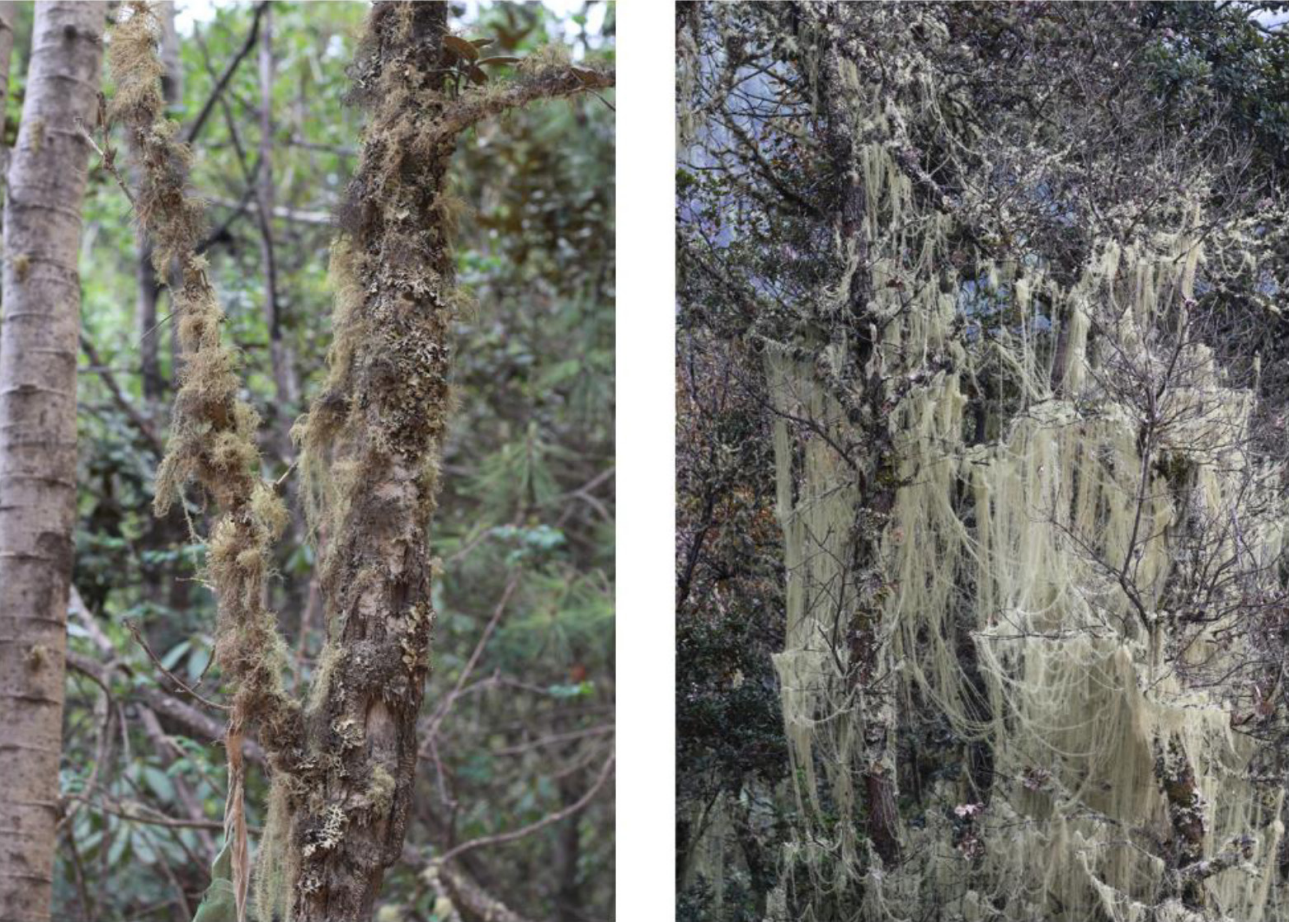
*Bryoria* spp. (left) and *U. longissima* (right).

The feeding habits of the monkeys were observed and recorded using a Leica 77 telescope (Solms, Germany) with instantaneous scanning sampling at 10‐min intervals. From May 2008 to April 2009 and September 2015 to August 2016, we tracked the monkeys of different groups in the morning, from when they left the sleeping site to when they entered the sleeping place in the afternoon. We followed each individual for at least 5 s to record which dietary items—fruits, seeds, insects, flows, and which lichen species—were consumed by the monkeys: (1) lichens (*Bryoria* spp. and *U. longissima*), which are significantly morphologically distinguishable (Figure 1). *Bryoria* spp. mainly covers tree trunks, with black colors, while *U. longissima* is principally intertwined among tree branches with suspending thread‐like white fibers; (2) dicotyledonous leaves and buds; (3) mature leaves (including bamboo leaves); (4) flowers; (5) fruits and seeds; (6) insects; (7) others, with discriminating methods described by Huang et al. (2017), at least 5 days or 50 h of eating data were recorded every month for each of the groups.

### 2.1 Laboratory work

From July to November 2015, between 11 and 16 samples of *Bryoria* spp. and *U. longissima* were collected randomly at different altitudes (2450–3450 m) of the five study sites. Each sample was classified and analyzed for its nutrient and chemical contents, including crude protein (CP), fat, all fiber fractions [neutral detergent fiber (NDF), acid detergent fiber (ADF), and acid detergent lignin (ADL)], ash, water‐soluble carbohydrate (WSC), and starch (Hou et al., 2018).

We first determined the physical differentiation between the two lichen species consumed, then gauged each nutritional (CP, fat, all fiber fractions, ash, WSC, and starch) or chemical (tannin) component (percentage) contained in each species. All components were measured with dry lichens. The chemical component, tannin, was determined in the Folin‐Ciocalteau phenol reagent (Box, 1983). The average of all samples was used in the calculation.

The food availability index (FAI), which measures the relationship between food supply and population structure (Hutto, 1990; Ritzema et al., 2017), was used to assess the relative abundance of two lichen groups. We calculated this index from June 2008 to September 2009 (Huang et al., 2012).

### 2.2 Data analysis

The following procedures were used to determine the amount of each component consumed by the monkeys from FBF, either from *U. longissima* or *Bryoria* spp.: total nonstructural carbohydrates (TNC) and energy were calculated (Hou et al., 2018). The energy value of NDF was estimated using a previously published NDF digestibility coefficient of 91% (Kirkpatrick et al., 2001).

A ratio was created for each lichen species in FBF:

$$R_{\left(U.\;\text{longissima}\right)}=\mathrm{Ua}/\left(\mathrm{Ua}+\mathrm{Ba}\right)$$

$$R_{\left(\text{Bryoria}\;\mathrm{spp}.\right)}=\mathrm{Ba}/\left(\mathrm{Ua}+\mathrm{Ba}\right)$$

where **a** is the physical proportion of *U. longissima* or *Bryoria* spp. in FBF, recorded from the wild; Ua is the physical proportion of *U. longissima*; and Ba is the same proportion of *Bryoria* spp.

The actual amount of a given component consumed from each of the lichen species from FBF was calculated with the following formula:

$$\mathrm{AU}=R_{\left(U.\;\text{longissima}\right)}\times U\%,\text{from}\;U.\;\text{longissima}.$$

$$\mathrm{AB}=R_{\left(\text{Bryoria}\;\mathrm{spp}.\right)}\times B\%,\text{from}\;\text{Bryoria}\;\mathrm{spp}.$$

where U% is the component percentage contained in *U. longissima*, and B% is the component percentage included in *Bryoria* spp. They were assessed in the laboratory.

Thus, the total amount regarding a specific component from both lichen groups is:

SUM=AU+AB.

For example, if FBF consists of 67% of *U. longissima*, and 25% of *Bryoria* spp., recorded from the field, *R*_(*U. longissima*)_ = Ua/(Ua + Ba) = 67/(67 + 25) = 0.73; *R*_(*Bryoria* spp.)_ = Ub/(Ua + Ba) = 25/(67 + 25) = 0.27. If component tannin in *U. longissima* is 4.5% and 6.5% in *Bryoria* spp., separately, from the laboratory records, the actual amount of tannin in FBF from *U. longissima* = 0.045 × 0.73 = 3.29%; and that from *Bryoria* spp. = 0.065 × 0.27 = 1.76%.

Separately, *R. bieti* consumes 3.29% and 1.76% of tannin from *U. longissima* and *Bryoria* spp., with a total of 5.05% from the lichens.

We assessed the relative abundance of two lichen groups (FAI) with the Mann‐Whitney *U*‐test to compare the time difference of feeding on *Bryoria* spp. to that on *U. longissima* and analyze the differences in components. Data were processed by SPSS 21.0, and the significance level was set to *p* < .05. All the results are shown as mean ± SD.

## 3 RESULTS

The first result of the study is the dietary components of *R. bieti*, which are summarized in the five groups. This monkey species feeds 78.9% of lichens, 4.0% of buds and young leaves, 5.1% of mature leaves, 7.3% of fruits, 1.5% of flowers, 1.9% of insects, and 1.3% of others. As for the lichen amounts, there is a significant difference between *Bryoria* spp. and *U. longissima* (*U* < .001, *n*1 = *n*2 = 24, *p* < .001)—a higher proportion of *Bryoria* spp. (64.63% ± 13.28%, and 69.86% ± 10.93%) than *U. longissima* (14.48% ± 12.12%, and 17.11% ± 11.56%). A year‐over‐year variation of the lichen consumption and a comparison between the two species collected from the five groups are provided in Table 1. The results indicate that the year variation of lichens consumed by the monkeys did not reach a significant level for each lichen species.

**TABLE 1:** Time budgets on different lichen species by *R. bieti*.

Period	Time percent spent on lichens (%)	
	*Bryoria* spp. (*n*)	*U. longissima* (*n*)
2008 May	63.2 (382)	1.0 (6)
2008 June	56.6 (179)	13.6 (43)
2008 July	76.0 (336)	7.9 (35)
2008 August	62.0 (631)	10.2 (104)
2009 September	61.4 (766)	5.2 (65)
2008 October	47.0 (316)	35.8 (241)
2008 November	54.5 (1397)	33.7 (865)
2008 December	87.3 (1442)	9.6 (159)
2009 January	91.0 (1676)	3.1 (57)
2009 February	60.5 (1049)	27.8 (482)
2009 March	57.7 (1823)	20.8 (658)
2009 April	58.4 (884)	5.0 (72)
2008–2009 monthly mean ± SD	64.63 ± 13.28	14.48 ± 12.12
2015 September	56.0 (981)	27.5 (481)
2015 October	50.3 (971)	38.5 (743)
2015 November	74.2 (1888)	15.8 (402)
2015 December	67.8 (1938)	28.8 (823)
2016 January	88.6 (2650)	9.0 (268)
2016 February	77.5 (1677)	2.6 (58)
2016 March	79.1 (1619)	10.4 (213)
2016 April	64.9 (2643)	11.3 (459)
2016 May	73.2 (1425)	3.2 (63)
2016 June	70.3 (1116)	11.7 (186)
2016 July	77.7 (243)	15.7 (69)
2016 August	58.7 (269)	30.8 (141)
2015–2016 monthly mean ± SD	69.86 ± 10.93	17.11 ± 11.56

**FIGURE 2:**
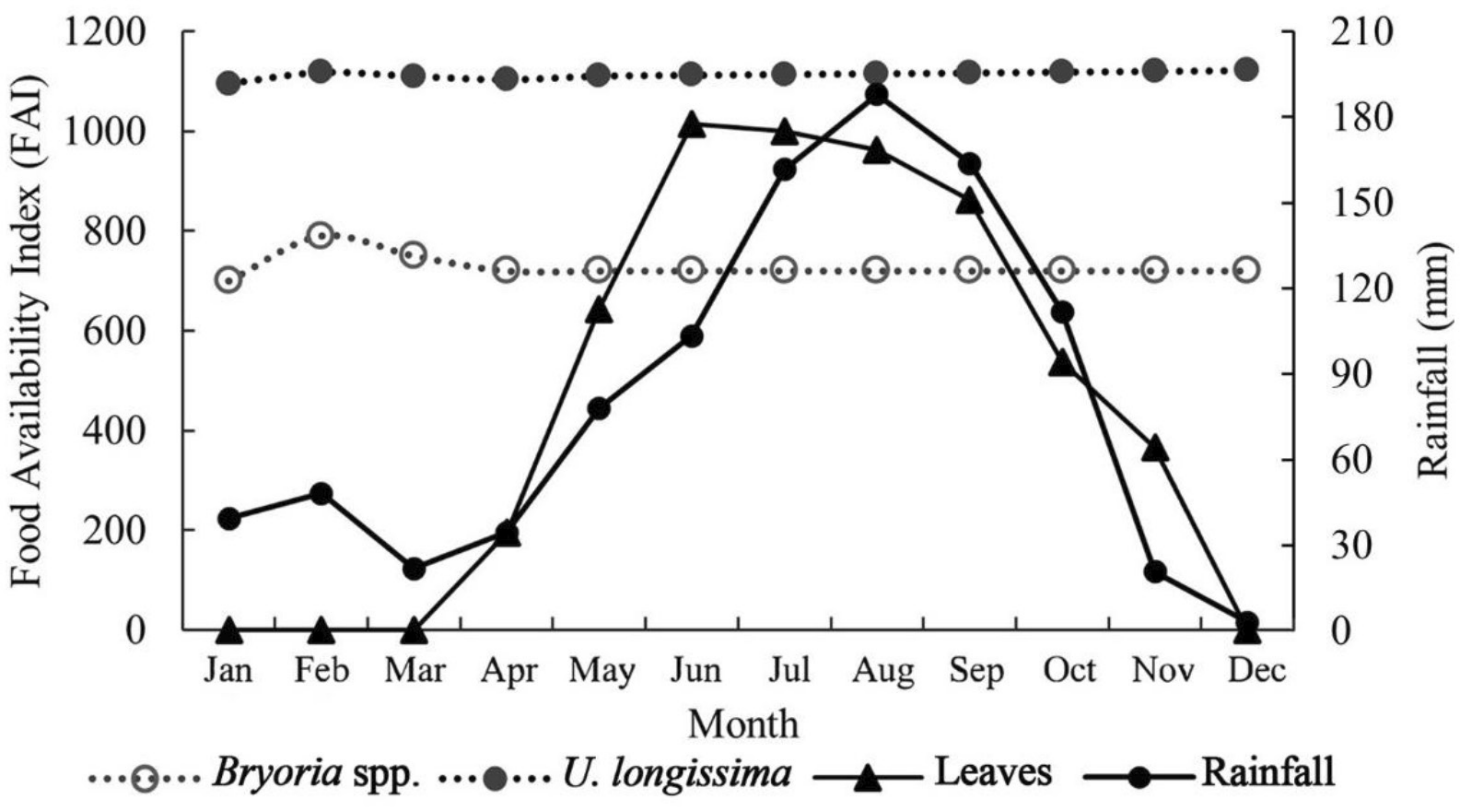
FAI index of the two lichen species, leaf variation and the rainfall records during the study period.

Concerning the FAI, both lichen species show a stable supply all year round, except for a lower *Bryoria* spp. in January. Leaves present a remarkable seasonal variation and extreme scarcity from December to April (dry season, Figure 2).

**FIGURE 3:**
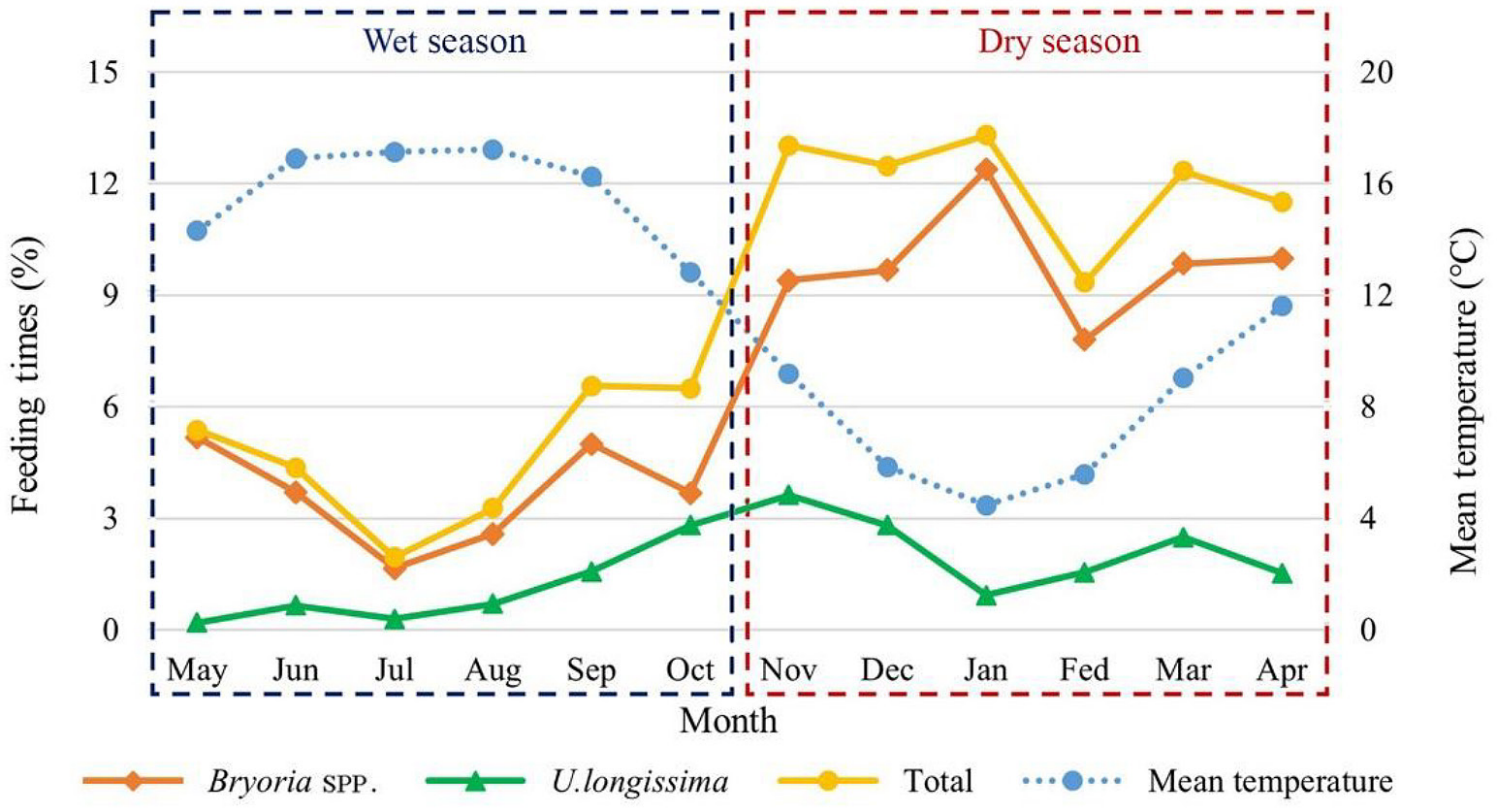
The monthly contributing proportion of two lichen species in different seasons.

When the two lichen species are combined, the difference between dry and wet seasons is illustrated in Figure 3. The dry season shows a significantly higher amount than the wet season. When they are analyzed separately, *Bryoria* spp. presents a significantly higher physical amount in the dry season than the wet one, but not *U. longissima*.

### 3.1 Lichen consumption

Monthly lichen consumption is compared between five *R. bieti* groups from the southernmost to northernmost of the monkey's distribution range (Table 2).

**TABLE 2:** Seasonal and regional lichen variations in the diet of *R. bieti*.^a^

Location	Feeding times (%)												References
	J	F	M	A	M	J	J	A	S	O	N	D	
Mt. Lasha	96	84	84	70	70	76	89	81	75	86	89	97	Our study
Samage	83	90	83	42	29	68	96	46	64	54	79	97	Grueter, Li, Ren, Wei, Xiang, and van Schaik (2009)
Tachen	74	82	48	40	44	59	54	48	54	55	61	69	Ding and Zhao (2004)
Wuyapiya	–	–	–	–	53	64	92	94	60	93	86	95	Kirkpatrick (1996)
Xiaochangdu	96	98	88	81	53	56	83	84	–	91	97	97	Xiang et al. (2007)

^a^ From J to D: January, February, March, April, May, June, July, August, September, October, November, and December.

Lichens are consumed all year around and account for 72.99 ± 11.30% (range from 56.27% to 82.70%) among five monkey groups, including the southernmost (82% for Mt. Lasha), midland (67% for Samage, 56% for Tachen, 77% for Wuyapiya), and northernmost (83% for Xiaochangdu) of the whole home range. The average monthly consumption is 72.44 ± 18.40% (range from 29% to 98%).

### 3.2 Nutritional and chemical components of the lichens

*Bryoria* spp. comprises significantly higher percentages of ash than *U. longissima*; the latter, however, includes significantly higher portions of ADF, fat, tannin, and energy than the former. After being adjusted, except for fat, monkeys consume a significantly higher amount of *Bryoria* spp. than from *U. longissima* (AU, Table 3).

**TABLE 3:** Nutritional and chemical components contained in the two lichen species and the amount consumed by *R. bieti* through FBF.

Nutrition type	Contained in lichens			AB and AU		
	*Bryoria* spp.	*U. Longissima*	*p*	*Bryoria* spp.	*U. Longissima*	*p*
Crude protein	5.36%	6.37%	*NS*.	4.34%	1.22%	**
Fat	1.02%	3.84%	**	0.83%	0.74%	*NS*.
Neutral detergent fiber	40.64%	34.58%	*	32.85%	6.62%	**
Acid detergent fiber	4.02%	5.90%	*	3.25%	1.13%	**
Acid detergent lignin	0.54%	0.42%	*NS*.	0.43%	0.08%	*
Ash	2.41%	1.78%	*	1.95%	0.34%	**
Water‐soluble carbohydrate	3.68%	4.05%	*NS*.	2.97%	0.78%	**
Starch	21.31%	19.78%	*NS*.	17.23%	3.78%	**
Tannin	0.26%	0.45%	**	0.21%	0.09%	**
Total non‐structural carbohydrates	50.56%	53.43%	*NS*.	40.88%	10.23%	**
Energy	13.85	14.86	**	11.20	2.85	**

*Note*: *U*‐text is used in Table 3, and * for *p* < .05, ** for *p* < .001.

### 3.3 Nutritional and chemical components between the dry and wet seasons

**FIGURE 4:**
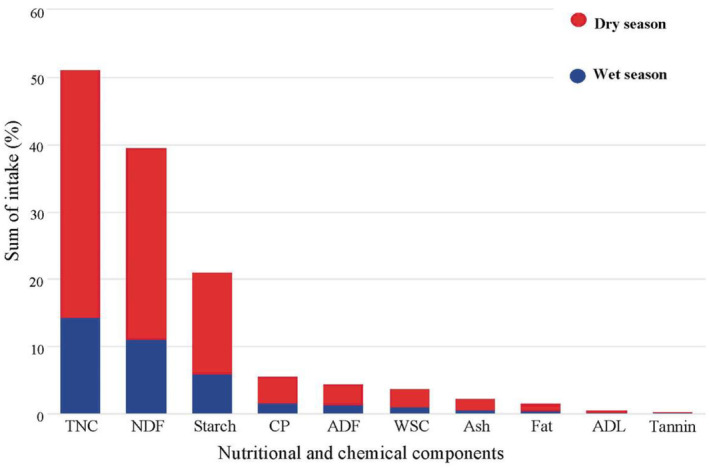
The nutritional and chemical components consumed by *R. bieti*, between dry and wet seasons, based on SUM values.

Lichens provide more significant components in the dry season than in the wet one (Figure 4). A general decreasing trend of nutritional and mechanical components was found: TNC > NDF > starch > CP > ADF > WSC > ash > fat > ADL > tannin.

## 4 DISCUSSION

This study exposes the uniqueness of the dietary selection of *R. bieti*, which considerably depends on lichens as a staple FBF and for their nutritional and chemical components. As expected, it provides valuable evidence and information to understand *R. bieti'*s unique ecological selection, environmental adaptation, and evolutionary development, shaped by the most frigid habitats of nonhuman primates at the highest altitude.

### 4.1 Unique dietary selection and evolutionary development

**FIGURE 5:**
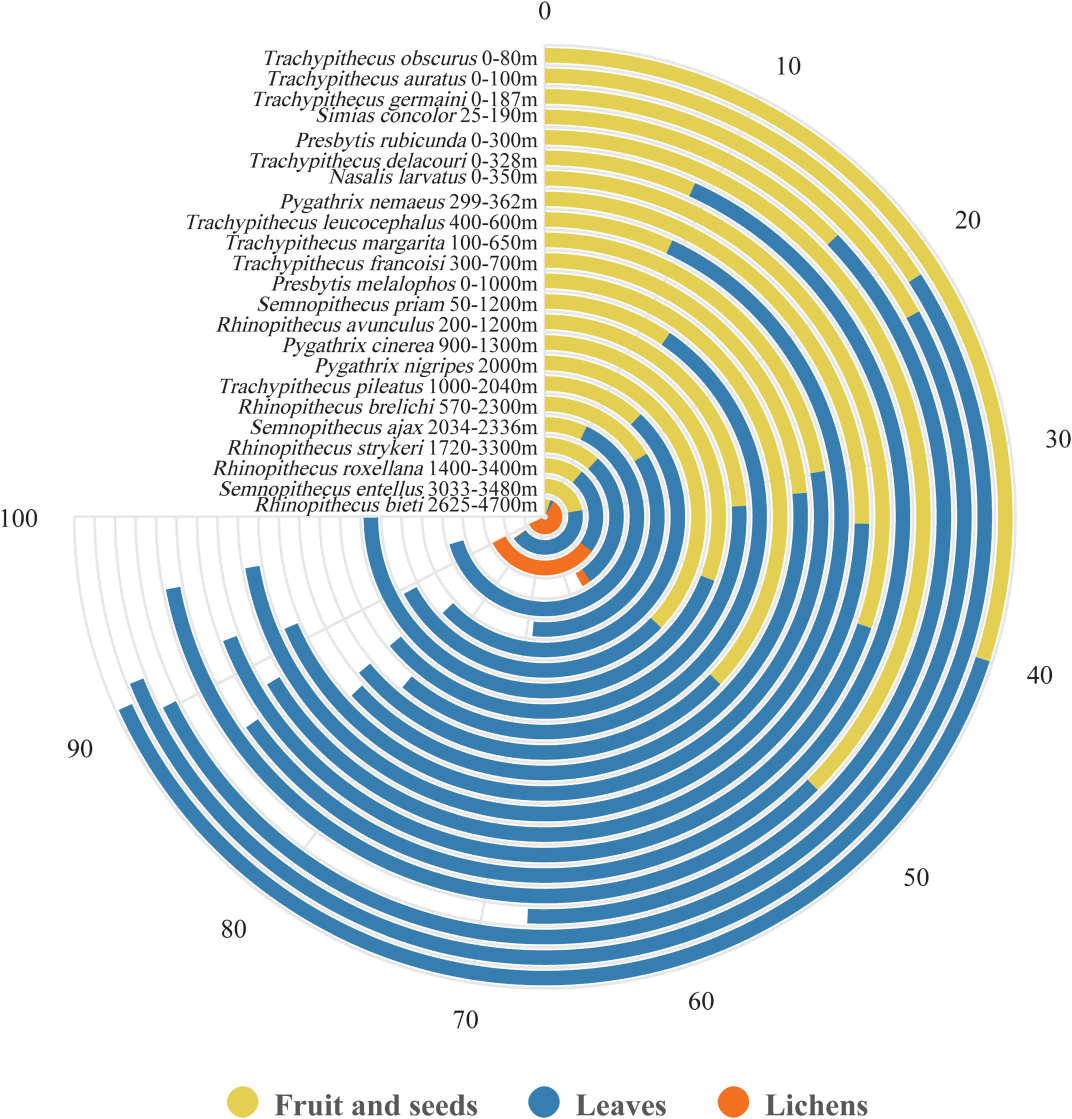
Dietary selection among Asian colobines. Database resources: Appendix Table A2.

According to its evolutionary development, the unique dietary selection of *R. bieti* seemed to develop during the Quaternary since they settled down in Southern East Asia during the Pliocene after a long journey from Africa to East Asia via Europe during the Miocene–Pliocene (Roos et al., 2011). Its dietary choice comparison with the other Asian colobines based on a broader literature review is provided in Figure 5.

*R. bieti* is singled out for its unique dietary selection—the highest lichen and the lowest leaf, fruit, and seed proportions compared with other Asian colobines. That can be regarded as a specific evolutionary development and the solution of environmental adaptation and ecological selection in the highest habitat elevation and harshest survival conditions of any other nonhuman primates on this globe (Kirkpatrick, 1996). Such scenarios imply that lichens might be an exceptional dietary choice for the ancestors of the snob‐nosed monkeys before they dispersed to the places where they dwell (Roos et al., 2011) (Figures 2, 3, and 5).

*Rhinopithecus bieti* and other Asian colobines originated from Africa in the Middle Miocene and migrated into Eurasia through the gateway of North Africa, possibly in the Late Miocene, about 10 mya ago (Davies & Oates, 1994; Roos et al., 2011). They arrived at a Convergency‐Divergency Center where *R. bieti* is located (Zhang et al., 2022). The oldest colobine fossil species associated with European and Asian taxa, *Mesopithecus*, unearthed in the Late Miocene (Davies & Oates, 1994; Heintz et al., 1981), was discovered recently in Zhaotong, Yunnan, in the deposit of the Late Miocene or Early Pliocene. It has been reckoned to be closely related to the taxa in *Rhinopithecus*, possibly *R. bieti* (Ji et al., 2020).

In other words, *R. bieti* is the only Asian colobine species located in the Qinghai‐Tibet Plateaus and Mt. Hengduan, which have experienced significant tectonic changes following the accelerated uplift of the plateau and mountains (Sun et al., 2011). Other colobines, however, dispersed and radiated to other parts and are widely distributed in East Asia and South East Asia, reaching coastlines (Peng et al., 1993; Zhang et al., 2022). Thus, *R. bieti*, different from other primate species in other parts of the world, can be regarded as a species facing severe challenges year‐round, divided into dry and wet seasons. It keeps the original dietary selection of the Asian snub‐nosed colobines—lichens, especially from *Bryoria* spp. (Figure 3). This also implies that, following a decrease in temperature in the dry season, *R. bieti* consumes more food and energy to maintain body temperature (Bissell, 2014). Such mechanisms are the adaptation of going through the period with the scarcest resources in the high altitudinal region.

Another interesting comparison is between *R. bieti* and other taxa in the same genus: (1) not all species of *Rhinopithecus* have FBF; and (2) not all *Rhinopithecus* species choose lichens as FBF. Among five taxa, lichen FBF has been reported for *R. bieti* (Grueter, Li, Ren, Wei, Xiang, & van Schaik, 2009; Kirkpatrick, 1996), *R. roxellana* (Hou et al., 2018; Li, 2006), and *R. strykeri* populations on Mt. Gaoligong in China (Yang et al., 2019). Another snub‐nosed monkey species, *R. brelichi*, found in Mt. Fanjing, Guizhou, has the FBF primarily including the buds. Thus, the taxa with lichen FBF are in China's higher mountains (Mts, Hengduan) and plateaus (the Qinhai‐Tibet and Loess linking the Qinling). Such scenarios must be relevant to the unique geographic and habitat choices after settling in East Asia under natural selection and environmental adaptation regulations and principles. Populations of *R. strykeri* in Northern Myanmar have a lower altitude (1720 m), and *R. avunculus* is primarily located in the forests of limestone mountains in northern Vietnam (Boonratana & Canh, 1998), which are quite different from those adopted by *R. bieti*, *R. roxellana*, and the populations of *R. trykeri* on Mt. Gaoligong in China (Yang et al., 2016).

### 4.2 Staple FBF of *R. bieti*

What is indicated by Table 2 shows that lichens are the most dominant food type of the monkeys analyzed (81.81 ± 8.32% of annual dietary consumption), and there is a stable supply among the five groups. Such a portion in the dry season can reach 100%. That is to say, lichens are the monkeys' dietary selection year‐round, which allows us to regard such selection as the staple FBF according to the definition proposed by Marshall and Wrangham (2007).

### 4.3 Nutritional character and ecological adaptation

Lichens have a crucial nutritional value for *R. bieti*, considering their dominant high proportion in annual dietary composition, especially in the dry season, and being regarded as FBF with more than 80.0% of dietary composition (Table 1). Some components, such as fibers, usually contain the elements of NDF, mainly cellulose, and hemicellulose, which are indigestible in normal animals (Lewis et al., 2005). However, *R. bieti* can hydrolyze them into digestible volatile fatty acids by unique microbial fermentative processes in their multichambered stomach (Li et al., 2023; Xia et al., 2022). Therefore, lichen with high NDF would be a thriving food and nutrient resource for *R. bieti*. Tannin is always avoided in food selection in primates since it is difficult to absorb (Chivers & Langer, 1994; DeGabriel et al., 2009). That may explain why *R. bieti* prefers *Bryoria* spp. over *U. longissima*, a selection of the low tannin level but higher NDF.

*Usnea longissima* contains significantly higher portions of fat, ADF, energy, and tannin than *Bryoria* spp.; the latter includes a considerably higher amount of DNF and ash (Table 3). However, after such numbers have been adjusted, referring to the actual amount consumed by *R. bieti* (SUM, including AB and AU), monkeys eat significantly higher proportions of *Bryoria* spp. rather than *U. longissima*, simply because the former occupies a much higher physical amount consumed (Table 3).

The study also reveals that *R. bieti* progressively reduces taking energy from TNC, NDF, starch, CP, ADF, WSC, ash, fat, and ADL, and the minimum amount of a chemical element (tannin, Figure 4). During the dry season, the creatures significantly consume more of those components from lichens, which, as addressed above, is due to the scarcity of other food resources in the season. That also implies that *R. bieti* receives more components from non‐FBF (fruits, seeds, and leaves) during the wet season (Figures 2 and 3). Thus, consuming lichens in the dry season, the only available food resource, is vital for *R. bieti* to cope with dual stresses: nutrition requirements and low temperature. Taking lichens, which have high availability and wide distribution in the dry season, is an evolutionary and ecological adaptation; avoiding monkeys consumes unnecessary energy loss due to traveling long distances to seek alternative foods. This energy‐conserving strategy is also very critical for other taxa in the genus in dealing with harsh ecology and unique periods; for instance, *R. roxellana* mainly consumes bark and buds in cold and food shortage periods (Hou, 2018; Hou et al., 2020), and Arctic fox (*Vulpes lagopus*) consumes stored food in cold winter (Prestrud, 1991).

### 4.4 The implication of conservation

In this study, lichens play a vital food supply for survivability and future development for *R. bieti* and the other two species in the same genus (Figure 5)—a unique evolutionary development, ecological selection, and environmental adaptation. Among them, *R. bieti* faces a remarkable conservation challenge due to the significantly declining lichen quality, production, and fragmentation (Grueter, Li, Ren, Wei, & van Schaik, 2009; Grueter, Li, Ren, Wei, Xiang, & van Schaik, 2009). It has been reported that a renewal cycle of lichens, if damaged, could take as long as 21 years (Kirkpatrick, 1996; Seaward, 1987). The significant impacts on lichens include:

1. Over the decades, human‐induced activities have prominently increased through deforestation, overgrazing, and cropland reclamation, which has caused soil erosion and rocky desertification. Such situations in the mountains where the species is located have become more severe since the 1990s, resulting in a sharp decline in biodiversity, frequent natural disasters, and severe ecological degradation. They have severely impacted lichens' quality and fragmentation (Jiang et al., 2003; Shang et al., 2018; Yin et al., 2020), and triggered significant natural resource exploitation, land conversion, and pasture extension, which have led *R. bieti*, like any other primate taxa, to face prominent conservation pressure in China (Pan et al., 2016; Xiao et al., 2003), especially suffering from the fragmentation of the lichens, soil erosion, and other ecosystems (Grueter, Li, Ren, Wei, Xiang, & van Schaik, 2009; Marshall et al., 2009).
2. The increasing air pollution has negatively impacted the growth and survivability of lichens in the regions (Cao, 1989). The Mts.Hengduan, where the studied species dwells, are considered one of the essential regions of carbon sinks in South and Southwestern Eastern Asia. Thus, the ecosystems of many animals and plants are susceptible to air pollution caused by human‐induced activities and natural modifications (Huang et al., 2020; Yin et al., 2020). Lichen is very sensitive to the environment and ecology, especially to atmospheric nitrogen (N) deposition that has increased in recent years in China, which can significantly disturb the nutrient balance of the lichens besides constraining their growth and survival. The lichens' thallus growth and propagule survival can substantially decrease when nitrogen addition changes from 6.25 to 50.0 kg N·ha^−1^ year^−1^. Further, lichen biomass could be reduced by 11.2%–70.2% when the deposition addition exceeded 6.25 kg N·ha^−1^ year^−1^ (Yin et al., 2020).
3. Climate change has placed another prominent pressure on lichen production. Evolutionarily, lichens appeared in about 600 mya (Yuan et al., 2005). However, they have changed significantly due to climate modifications from icehouse to greenhouse conditions, particularly during the Quaternary glacial and interglacial cycles (Ellis, 2019). Research indicates that from 1954 to 2009, the annual temperature in the region (Mts. Hengudan) increased by 0.9°C and the annual precipitation decreased by 185 mm (Chen et al., 2012). Consequently, lichens and many other non‐vascular epiphyte species are heavily negatively impacted due to their very high sensitivity to temperature and moisture following climate change (Song et al., 2012).

Thus, the results of this study indicate that this and the other two species of snub‐nosed monkeys depend essentially on lichens, as FBF are facing prominent challenges due to the damages and reduction of the lichens that are, however, most vulnerable to climate change, air pollution, and human activities. Increasing attention to the protection of lichens is critical to mitigate conservation pressures on the *R. bieti*.

The results found in this study can provide a scientific reference to maintaining balanced nutritional and chemical components for the monkeys, which have developed during their evolutionary and phylogenetic progress and are the consequence of ecological adaptation. Since this primate species has been categorized as a critically endangered primate species, captivity‐feeding programs have been applied in some places (He & You, 2015). Alternative food resources have been used in the case of lichens being absent. Thus, it is critical to maintain their nutritional and chemical balances by referring to Figure 4 to select alternative dietary components and plant species for captivity breeding.

## AUTHOR CONTRIBUTIONS

**Hao Pan:** Writing – original draft (lead); writing – review and editing (lead). **Rong Hou:** Formal analysis (lead). **He Zhang:** Formal analysis (supporting); writing – original draft (supporting). **Yanpeng Li:** Conceptualization (equal); investigation (equal). **Zhipang Huang:** Conceptualization (equal); funding acquisition (equal); investigation (equal); project administration (supporting). **Liangwei Cui:** Funding acquisition (equal); project administration (supporting). **Wen Xiao:** Funding acquisition (equal); project administration (supporting).

## FUNDING INFORMATION

This study was sponsored by the National Natural Science Foundation of China (Grant no: 31860168, 31860164, 32170507), the Postdoctoral Fellowship Program of China Postdoctoral Science Foundation (GZC202300995), the Local Universities Union Project of Yunnan Province Natural Science Foundation (202001BA070001‐227), the Ten‐thousand talent plan of Yunnan Province (YNWR‐CYJS‐2018‐052; YNWR‐QNBJ‐2019‐262), and The Project for Talent and Platform of Science and Technology in Yunnan Province Science and Technology Department (202105AM070008).

## CONFLICT OF INTEREST STATEMENT

The authors have no conflict of interest to declare.

## Data Availability

The datasets generated during and/or analyzed during the current study are available in the figshare repository (10.6084/m9.figshare.22785356).

## Appendix

**TABLE A1:** Food repertoire of *R. bieti* at Mt. Lasha.

Families	Genus	Species	Food parts
Actinidiaceae	*Actinidia*	*purpurea*	bud, leaf, flower, fruit
Adoxaceae	*Viburnum*	*betulifolium*	fruit
Aquifoliaceae	*Ilex*	*delavayi*	leaf
Araliaceae	*Acanthopanax*	*evodiaefolius*	bud, leaf, flower, fruit
Betulaceae	*Betula*	*albo*	bud, leaf
Caprifoliaceae	*Lonicera*	*hispida*	bud, leaf
Celastraceae	*Euonymus*	*tingens*	fruit
Clethraceae	*Clethra*	*monostachya*	bud, leaf
	*Cornus*	*controversa*	fruit
Cornaceae	*Macrocarpium*	*chinense*	flower, fruit
Helwingia	*Helwingia*	*japonica*	bud, fruit
Lauraceae	*Lindera*	*cubeba*	bud, flower
Liliaceae	*smilax*	*menispermoidea*	bud, fruit
Magnoliaceae	*Schisandra*	*rubriflora*	bud, leaf, flower, fruit
Ranunculaceae	*Clematis*	*Yunnanensis*	bud, leaf, flower
Rosaceae	*Prunus*	*conadenia*	bud, leaf, flower, fruit
	*Sorbus*	*rufopilosa*	bud, leaf, flower, fruit
		*thibetica*	bud, leaf, flower, fruit
		*zahlbruckneri*	bud, flower, fruit
		*megalocarpa*	bud, leaf, flower, fruit
		*oligodonta*	bud, leaf, flower, fruit
Salicaceae	*Populus*	*bonatii*	bud, leaf
		*yunnanensis*	bud, leaf, flower
	*Pterocarya*	*delavayi*	bud, leaf, flower, fruit
	*Salix*	*radinostachya*	flower
Sapindaceae	*Acer*	*cappadocicum*	bud, leaf
		*flabellatum*	bud, leaf
		*oliverianum Pax*	bud, leaf
symplocaceae	*Symplocoa*	*grandia*	leaf, flower

**TABLE A2:** Data sources of dietary selection among Asian colobines.

Genus	Species	References
*Nasalis*	*larvatus*	Yeager (1989), Rowe and Myers (2016)
*Presbytis*	*melalophos*	Davies and Baillie (1988), Nijman et al. (2020)
	*rubicunda*	Hanya and Bernard (2012)
*Pygathrix*	*cinerea*	Long (2020), Long, Duc, et al. (2020)
	*nemaeus*	Phiapalath et al. (2011)
	*nigripes*	Rawson (2009), Roos et al. (2011)
*Rhinopithecus*	*avunculus*	Rowe and Myers (2016), Hai (2012)
	*bieti*	Present study; Rowe and Myers (2016)
	*brelichi*	Xiang et al. (2013), Rowe and Myers (2016)
	*roxellana*	Li (2007), Rowe and Myers (2016)
	*strykeri*	Yang et al. (2019)
*Semnopithecus*	*ajax*	Thakur et al. (2022)
	*entellus*	Sayers and Norconk (2008)
	*priam*	Vanaraj and Pragasan (2021), Singh et al. (2020)
*Simias*	*concolor*	Erb et al. (2012)
*Trachypithecus*	*auratus*	Tsuji et al. (2019)
	*delacouri*	Workman (2010)
	*francoisi*	Zhou et al. (2006)
	*germaini*	Le et al. (2019)
	*leucocephalus*	Zhou et al. (2011)
	*margarita*	Hoang et al. (2022)
	*obscurus*	Ruslin et al. (2019)
	*pileatus*	Solanki et al. (2008)
